# Spatiotemporal trends and multilevel determinants of coexisting maternal and child undernutrition in Nepal: evidence from Nepal Demographic and Health Survey 2006–2022

**DOI:** 10.1186/s40795-026-01452-8

**Published:** 2026-08-07

**Authors:** Rubina Karki, George Chikondi Samu, Dziko Nelson Ntaba, John Mugisha, Noreen Clara Ng’ambi, Sellina Samu, Bishnu Kumar Karki

**Affiliations:** 1 Department of Nutrition and Dietetics, CAFODAT College, Faculty of Science and Technology, Purbanchal University, Kathmandu, Nepal; 2 School of Public Health, Department of Nutrition and Food Hygiene, Southern Medical University, Guangzhou, China; 3 SureCare Medical Supplies, Lilongwe, Malawi; 4 Department of Agricultural Research Services, Lilongwe, Malawi; 5 Rwanda Implementation Science and Health Systems Lab, Kigali, Rwanda; 6 MASM Medi Clinics, Lilongwe, Malawi; 7 Ministry of Home Affairs, Government of Nepal, Kathmandu, Nepal

**Keywords:** Coexisting maternal and child undernutrition, Stunting, Wasting, Underweight, Spatial analysis, Hotspot analysis, Nepal

## Abstract

**Background:**

Coexisting maternal and child undernutrition (CMCU) is a critical aspect of intergenerational malnutrition. The evidence on its epidemiological trends and spatial distribution remains limited in Nepal. This study investigated the temporal trends, determinants, and spatial distribution of CMCU in Nepal from 2006 to 2022.

**Method:**

We used data from the 2006, 2011, 2016, and 2022 Nepal Demographic and Health Survey (NDHS) databases. Maternal and child pairs with complete anthropometric records were included in the analysis. The co-occurrence of maternal undernutrition (height < 145 cm) and child stunting/wasting/underweight were defined as CMCU. Survey-weighted descriptive analysis was performed to assess prevalence trends. Individual- and community-level determinants of CMCU were assessed using multilevel logistic regression with a two-level hierarchy. Individual children and their households (level one) were grouped within community enumeration areas or clusters (level two). Global Moran’s I and Getis-Ord Gi* Hotspot analysis were performed to assess spatial autocorrelation and local clustering, respectively.

**Results:**

The prevalence of CMCU decreased from 9.78% in 2006 to 4.68% in 2022, a 52.1% decrease over 16 years). A PCV of 0.802 in Model IV of the community level variation has been explained after adding both the individual and community factors. Even low ICC = 0.029 in Model IV shows that only 2.9% of the remaining, unexplained variances in Child and Maternal Co-undernutrition (CMCU) is caused by unmeasured community clustering factors as compared to ICC = 0.134 in model I which shows that 13.4% of the variance is explained by unobserved community level factors. This means Model IV shows that 97.1% variance is explained by individual and household level factors.

CMCU odds were inversely associated with maternal education (AOR = 0.68(95% CI: 0.50–0.91), 0.39(95% CI: 0.26–0.58), 0.20(95% CI: 0.07–0.58) for primary, secondary, higher respectively relative to no education). Children aged 12–59 months had higher CMCU odds than < 12 months. Odds were lower in contraceptive users (AOR = 0.75(95% CI: 0.60–0.93)) and higher for perceived health facility distance (AOR = 1.24(95% CI: 1.00–1.52)). The spatial average was significantly clustered in 2022 (Moran’s I = 0.058, Z = 2.548) with hotspots in the southern Terai.

**Conclusion:**

Despite an overall decline in CMCU during 2006–2022, socioeconomic and regional inequalities were observed in Nepal. CMCU was associated with maternal education, child age, reproductive health services utilization and access of health services. The new trend of spatial clustering in the southern provinces makes it important to develop geographically-targeted and equity-sensitive approaches to aid the implementation of multisectoral nutrition strategies in Nepal.

**Supplementary Information:**

The online version contains supplementary material available at https://doi.org/10.1186/s40795-026-01452-8.

## Background

Malnutrition is one of the highest causes of morbidity and mortality among children under five years of age globally and is responsible for almost half of under-five mortalities [1]. The prevalence is particularly high in South Asia among under-five children, where the prevalence of stunting and wasting was 31.8% and 14.8% respectively [2]. Despite the successful stunting reduction (57% in 1996–25% in 2022) and underweight (43% in 1996–19% in 2022), Nepal remains highly afflicted by malnutrition [3]. The NDHS 2022 report shows that among Under-five children, stunting prevalence is 25%, wasting 8% and underweight 19% while BMI < 18.5 kg/m² is 16.2% among the reproductive age women [3].

The biological determinants of CMCU are related to the intergenerational cycle of malnutrition. Maternal short stature (< 145 cm) indicates chronic undernutrition during crucial growth years. It is associated with inadequate pelvic size, obstructed labour, low birth weight, and child stunting. Low birth weight is associated with childhood stunting, thus providing the link in the intergenerational cycle. Maternal nutrition also has direct implications for breast milk availability and quality, which is crucial for the first 6 months of life. Malnourished mothers may also have impaired care-giving abilities and frequent undernutrition is likely to lead to unresponsive feeding during the complementary period (6–23 months), when stunting prevalence is highest. The biological pathways and the common underlying determinants (poverty, food insecurity, poor sanitation) are the reasons why maternal and child under-nutrition usually co-exist in the same households.

The coexistence of maternal and child undernutrition (CMCU) is a potentially serious but less-studied occurrence of the intergenerational cycle of malnutrition. CMCU adversely affects child development outcomes through the combined effects of reduced maternal care capacity, poor breastfeeding and complementary feeding practices, and shared household deprivation [4]. The most recent assessments of the 2022 NDHS data revealed that among children aged 6–59 months, CMCU and anemia coexistence was 16.0%, and the strongest related factors were maternal education, family wealth, and women’s decision-making freedom [5]. However, CMCU has not been widely recognized as a prospective data outcome defined across several rounds of the survey, and its epidemiological profile has not been evaluated in detail for Nepal.

The available nationally representative literatures from Nepal have contributed immensely to the knowledge across different risks and associated characteristics of child undernutrition and spatial variation of under-nutrition, but have left a considerable gap in the coexisting under-nutrition character of mothers and children. A recent study identified maternal education, household wealth, and child age as persistent drivers of child stunting. However, it treated maternal and child under-nutrition as independent outcomes rather than coexisting household conditions. [6]. Another study found strong effects of maternal BMI and education on the nutritional outcomes of children, although this analysis did not pertain to dual maternal-child undernutrition co-existence [7]. Few studies have also assessed the double burden of malnutrition within households, where Aryal et al. recently assessed that in households with an overweight/obese mother, they also have undernourished child with a prevalence of 6.6% [8], while a multicountry comparison; identified maternal age and not practicing adequate breastfeeding as drivers for the double burden of malnutrition in Nepal [9]. These important findings bring a lot of knowledge about the nutrition transition, but they do not address the situation of concurrent maternal and child undernutrition, which reflects the deprivation at the household-level and is a key factor for transmission of intergenerational risk factors [10]. In terms of spatial analysis, development has been remarkable in the case of Nepal, where bayesian spatial modelling of NDHS 2016 highlighted the presence of significant geographic clustering for stunted and wasted children, which are also disproportionally observed in Lumbini, Karnali and Madhesh provinces [11], and province-wise trend studies have confirmed the evidence of geographic inequities [12, 13]. However, no study used spatial analysis to investigate the presence of geographic clustering for CMCU transmission and whether it has changed during the last years together with boundaries alterations in administrative regions of Nepal. This knowledge gap is overcome by the present study, which combines different CMCU-related considerations, including time series, shared influences and spatial analyses for 16 years.

The malnutrition in Nepal based on spatial characteristics also has inequities associated with it. The stunting prevalence is found to be significantly higher in the rural areas (31%) compared to urban areas (22%). Across the ecological zones, stunting is found to be significantly higher in mountainous region (42%) compared to terai (25%) and hilly (22%) regions [3]. Bayesian spatial modeling evidence also suggests that malnutrition, specifically stunting and wasting, are clustered in space both at zonal and district levels and these determinants are unstable spatiotemporal hot spots in the mid-western and far western development regions [11]. Tools for analyzing spatial data include Global Moran’s I or Getis-Ord Gi* hot-spot analysis which determine the presence of statistical significance in these spatial disease and malnutrition pattern based observations and determine significant hot spots and cold spots with statistically significant high or low prevalence [11].

However, there is limited knowledge of the spatiotemporal trends and determinants of CMCU. This research aims to assess Spatiotemporal trends and multilevel determinants of Coexisting Maternal and Child Undernutrition in Nepal, using Nepal DHS.

## Methodology

### Data source and study population

**Fig. 1:**
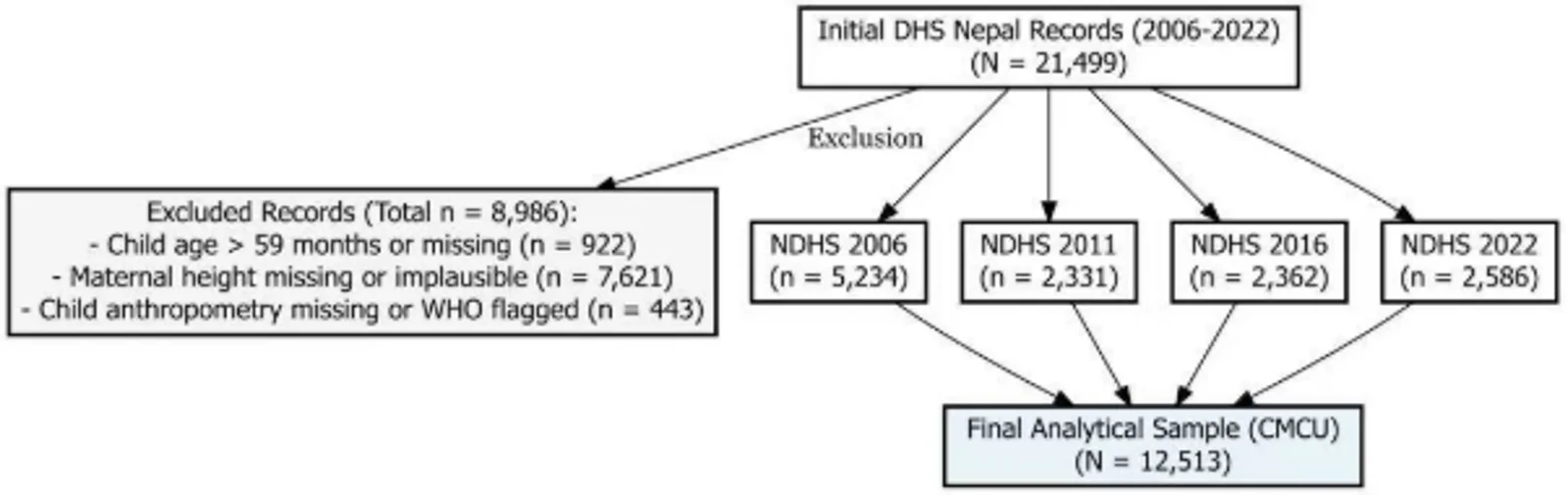
A schematic representation of the sampling procedure for the study on CMCU among mothers with children aged 0–59 months in Nepal, 2006–2022

The data were derived from the four rounds of the Nepal Demographic Health Survey (NDHS) conducted in 2006, 2011, 2016, and 2022. NDHS is a representative nationwide survey of households, is cross-sectional in nature and conducted by the Ministry of Health and Population with technical support from ICF International [14–18]. NDHS utilizes stratified two-stage cluster sampling technique where the Enumeration Areas (EAs) are selected with probability proportional to size and households are systematically selected from each EA [3]. The source population was all child-bearing women aged 15 years − 49 years in Nepal. The study population was selected from mother-child pairs from Nepal Demographic Health Surveys (NDHS) of 2006, 2011, 2016 and 2022, where the children were under five years of age and were living with their respective mothers which is shown in Fig. 1. Out of all pairs, only those with complete anthropometric measurements were selected, including maternal height and child anthropometric indicators of undernutrition based on Height-for-age (Stunting), Weight-for-age (Underweight), Weight-for-height (Wasting).

The mother-child pair were formed by linking all children born to a mother with the unique household ID and individual ID in the NDHS data files. For the mothers with multiple eligible children, we selected the youngest one to prevent the clustering of siblings and to ensure their statistical independence. This is in line with the analytical guidelines provided in the DHS reports. Each recode file for each year was cleaned first through individual structural standardization then the data for the whole four years was pooled together by vertically stacking the four survey years for each of the Children’s, Women’s and Household recodes. Then a horizontal merge was done by combining the three distinct files into one master data frame. Children recode file was used as the baseline then matched every single child to their mother using the case ID in the KR file. No siblings were deleted and all children were accounted for. To ensure statistical independence a Generalized Linear Mixed Effects model (glmer in R) which accounts for community-level random intercept (1|cluster_id) was used. The model explicitly recognizes that children are grouped inside communities and households. The statistical algorithm automatically adjusts the standard errors to account for these shared family connections. This ensures that our final Adjusted Odds Ratios (AORs) and 95% confidence intervals are entirely honest, valid, and fully corrected for family grouping.

The number of excluded records was provided at each stage of exclusion in Fig. 1.

#### Missing data

For each variable, the percentage of missing data was calculated. After combining children’s data from 2006 to 2022, 922 children were flagged as older than 59 months or had missing age data, 7,621 mothers had missing or biologically impossible height. After checking Children’s height and weight z-scores, 443 records show missing data or WHO error flag. Missing data were excluded using a two-stage approach whereby we prioritized maximum sample retention and eliminating selection bias. Out of our initial pool of 21,499 records from 2006 to 2022 survey years, we limited our data cleanup strictly to essential medical eligibility criteria to qualify for maternal and child undernutrition. We removed 8,986 records simply because they lacked the core physical measurements needed to calculate undernutrition in the first place specifically, invalid child age entries under five years ((*n* = 922), implausible maternal heights (*n* = 7,621), or incomplete child growth metrics (*n* = 443). We avoided a complete-case analysis that would have led to throwing away an entire family’s profile over a single skipped survey answer. Instead, we caught these questionnaire skips and blank spaces in our code and turned them into clear, descriptive categories (like labeling first-born children as “First Birth/Missing” since they naturally have no older sibling to calculate a birth interval from). This practical choice prevented any accidental data loss, keeping our final sample perfectly stable at 12,513 children.

### Outcome variables

In this study, the dependent variable is CMCU a binary variable coded as 1 when both the mother and child are undernourished, and 0 otherwise. Maternal undernutrition is defined as a height less than 145 cm [19, 20].Maternal height is a reliable indicator of long-term nutritional status and reflects the cumulative effects of childhood undernutrition, which may influence maternal and child health outcomes [21].While child undernutrition is identified by any anthropometric indicator height-for-age, weight-for-age, or weight-for-height below − 2 standard deviations from the WHO growth standards [22].

### Independent variables

The list of independent variables was derived from UNICEF’s conceptual framework for both maternal and child undernutrition and relevant studies. These were classified as individual and community-level determinants (Table 1).

**Table 1:** Individual and community level variables

Variables	Recoding
Individual-level variables	
Media exposure	0 = No, 1 = Yes
Cigarette smoking	0 = No, 1 = Yes
Toilet facility	0 = Unimproved, 1 = Improved
Contraceptive use	0 = Do not use, 1 = Use
Births in last five years	1 = < 3 births, 2 = ≥ 3 births
Parity	Continuous (number of children ever born)
Birth order	1 = < 3, 2 = ≥ 3
Birth interval	1 = < 36 months, 2 = ≥ 36 months
Type of birth	1 = Single, 2 = Multiple
Household size	1 = > 5 members, 2 = ≤ 5 members
Employment status	0 = Not employed, 1 = Employed
Pregnancy status	0 = Not pregnant, 1 = Pregnant
Partner age gap	1 = < 5 years, 2 = ≥ 5 years
Partner educational gap	0 = Same/Less, 1 = Mother more
Community Level variables	
Source of drinking water	0 = Non-piped, 1 = Piped
Electricity	0 = Not available, 1 = Available
Place of residence	1 = Urban, 2 = Rural
Administrative region (Province)	1 = Bagmati, 2 = Gandaki, 3 = Karnali, 4 = Koshi, 5 = Lumbini, 6 = Madhesh, 7 = Sudurpashchim
Community distance from health facility	0 = Not a big problem, 1 = Big problem
Community illiteracy level	0 = Low, 1 = High
Community poverty level	0 = Low, 1 = High

In order to ensure longitudinal harmonization, data from 2006 to 2022 were aligned according to transition of the country from the previous five Development Regions to seven Federal Provinces created by the Constitution promulgated in 2015. Since the surveys in 2006, 2011, and 2016 referenced the old regions, a mapping process from district to province was implemented to extrapolate past observations into current governance units. For each of the 75 districts from the old regional setup, current province affiliation was geographically assigned. The two districts that were made separate units after the 2015 reorganization, Nawalparasi and Rukum, were allocated historic data based on the leading present-day province administrative unit, Lumbini and Karnali, respectively. This process enabled the longitudinal graphing and statistical analysis of CMCU prevalence by province across survey waves, while also providing spatial analysis (Moran’s I and Gertis Ordi GI) that reflects present-day governance units.

Community-level factors were obtained by taking the individual responses of all the enumerated members in a particular enumeration area (EA). Community poverty status was derived from the percentage of households in the same EA belonging to the poorest or poorer wealth quintiles; EA with greater than 50% members in the poorest or poorer wealth quintile was named ‘high poverty’. Community illiteracy status was derived from the percentage of women in the EA who had no formal education; EA with greater than 50% women with no formal schooling was named ‘high illiteracy’. Community long distance from health facility were derived from the average number of respondents in the EA who reported long distance from the facility as a ‘big problem’ and EA with greater than 50% respondents who reported a ‘big’problem with distance from health facility was named ‘distance problems’. A cutoff point of 50% was used based on descriptive analysis of the individual EA values for each variable.

Generating these variables also followed a methodology by Bezie & Tesema, (2025). We wish to clarify that our variable construction strictly follows established, peer-reviewed demographic modeling methodologies. Because standard Demographic and Health Surveys (DHS) do not include pre-built contextual markers for clusters beyond region and residence type, we generated our community variables by aggregating individual household metrics at the cluster level (v001), stratified by survey wave year. To ensure complete transparency and reproducibility, our exact data-engineering methodology follows this established framework:

#### Direct community-level variables

Variables such as geographic province and urban/rural residence were utilized directly from the primary survey dataset without any manual manipulation.

#### Aggregated community-level variables

Contextual variables for Community Poverty and Community Illiteracy were built by calculating the continuous proportion of individual-level attributes within each cluster (i.e., the mean proportion of uneducated mothers and households in the bottom two wealth quintiles).

#### Dichotomization based on the national median

Because these aggregated cluster-level proportions were not normally distributed, they were categorized into binary factors (“High” vs.“Low”) based on their wave-specific national median values.

Clusters with proportions sitting strictly above the national median line were categorized as experiencing high illiteracy or high poverty, while those at or below the median served as the low-exposure reference groups.

Independent variables selection was done based on UNICEF conceptual framework of maternal and child undernutrition determinants, which divided causes into immediate, underlying and basic. Variables at the three levels were included to capture the entire causal pathway while preventing overadjustment. Potential mediators (contraceptive use, antenatal care) were retained in the model as confounder for the association between further upstream variables (education and wealth) and child malnutrition condition. It was noted that some of the variables (birth interval, birth order) in the model could be on the causal pathway of food insecurity to CMCU.

The following Directed Acyclic Graph (DAG) integrated as Supplementary Figure S1, fully aligned with the UNICEF Conceptual Framework on the Determinants of Nutrition.

### Statistical analysis

All analyses were conducted using the survey weights accounting for the complex survey design (stratification and clustering) with the survey package in R. Descriptive statistics are presented as weighted proportions with 95% confidence intervals (CIs). We fitted a two-level model. Accounting for the nested hierarchy of the survey data, individual children and their households (level one) were grouped within community enumeration areas or clusters which represented the second (level two). A two-level multilevel mixed-effects binary logistic regression model was used:

$$\mathrm{Yij}=\mathrm\beta0+\mathrm\beta1\mathrm X1\mathrm{ij}+\dots+\mathrm\beta\mathrm m\mathrm X\mathrm m\mathrm i\mathrm j+\varepsilon\mathrm{ij}+\mathrm U$$

Cluster-level variability was assessed where Model II included individual-level variables, Model III included community-level variables and Model IV included both. By utilizing this hierarchical block structure, Model IV successfully isolates the Controlled Direct Effects (CDE) of actionable public health entry points (such as improved toilets, media exposure, and contraceptive use). It tests whether these targeted interventions retain an independent protective capacity even when families are trapped within identical layers of macro-socioeconomic deprivation, providing clear, unconfounded evidence for framing national policy.

Model fit indices (AIC, BIC, log-likelihood) were used to determine overall model fit. The intraclass correlation coefficient (ICC) was used to determine the proportion of variation explained by differences between communities. The proportional change in variance (PCV) calculated the percentage reduction in community variation from the null model to the model of interest. The median odds ratio (MOR) represented the median odds ratio between the highest and lowest risk community for a randomly sampled pair with similar covariates. The generalized variance inflation factor (GVIF) was used to assess multicollinearity amongst the predictor variable in Model IV where a GVIF < 5 was considered acceptable [23]. Odds ratios (OR) and adjusted odds ratios (AOR) with 95% confidence intervals (CI) are provided. A p-value < 0.05 was considered statistically significant [24].

The Model IV was developed through backward elimination procedure. We began with a full model containing all variables, and continuously removed the candidate variable with the highest p-value at each step, until all remaining variables had a p-value < 0.20. The p-value cut-off of 0.20 was selected to balance a conservative approach to variable selection with the potential inclusion of important confounders that may be non-significant at conventional levels. At each step, likelihood ratio tests were used to assess nested models. All variables retained in Model IV are reported, irrespective of their significance in the final model, to enhance transparency regarding the adjustment set.

### Spatial analysis

Global spatial autocorrelation of CMCU prevalence for each survey round was assessed using the K-nearest neighbor weights matrix was used with a fixed threshold of (K = 8). This approach works by identifying the eight closest survey clusters to each individual cluster on the map. By calculating the straight-line distance between these points, the model builds a fair and balanced neighborhood map regardless of how crowded or sparse the region is.

Moran’s I in QGIS was used to determine spatial clustering for each of the 4 survey years. Moran’s, I value ranges from − 1 to 1. The value approaching (−1.0) signifies structural spatial dispersion, indicating that clusters of high and low CMCU rates alternate in a disjointed, regular pattern across neighboring units. Conversely, an index value approaching (+ 1.0) confirms significant spatial clustering, demonstrating that areas with elevated undernutrition rates are geographically close to other high-prevalence zones, whereas a value of zero denotes a completely random, non-patterned spatial distribution. A p value of < 0.05 was used to assess the significance of spatial pattern. Only 2022 survey data showed a lower p-value of < 0.05 showing that the observed spatial pattern is unlikely to be a random variation [25].

Getis Ord Gi was used to calculate spatial autocorrelation variations across the Nepal by determining the Gi* value for each region. Areas marked in red indicated high prevalence, while blue areas reflected lower prevalence rates [25].

### Missing data

The data cleaning and handling of missing values were executed through careful checking and rectifying of inconsistencies and discrepancies. A two-stage approach was used to handle missing values while prioritizing maximum sample retention and eliminating selection bias. Out of our initial pool of 21,499 records from 2006 to 2022 survey years, we limited our data cleanup strictly to essential medical eligibility criteria to qualify for maternal and child undernutrition. We removed 8,986 records simply because they lacked the core physical measurements needed to calculate undernutrition in the first place specifically, valid child age entries under five years (*n* = 922 were removed), plausible maternal heights (*n* = 7,621 were removed), or complete child growth metrics (*n* = 443 were removed). We avoided a complete-case analysis that would have led to throwing away an entire family’s profile over a single skipped survey answer. Instead, we caught these questionnaire skips and blank spaces in our code and turned them into clear, descriptive categories (like labeling first-born children as “First Birth/Missing” since they naturally have no older sibling to calculate a birth interval from). This practical choice prevented any accidental data loss, keeping our final sample perfectly stable at 12,513 children.

#### Ethical consideration

The data used for this study were secondary data that were publicly available and the the ICF International Institutional Review Board exempted from the requirement of individual consent. The study was conducted with the appropriate requests granted from the DHS Program. Data were assessed and analyzed ensuring participants’ confidentiality throughout the study.

## Results

**Fig. 2:**
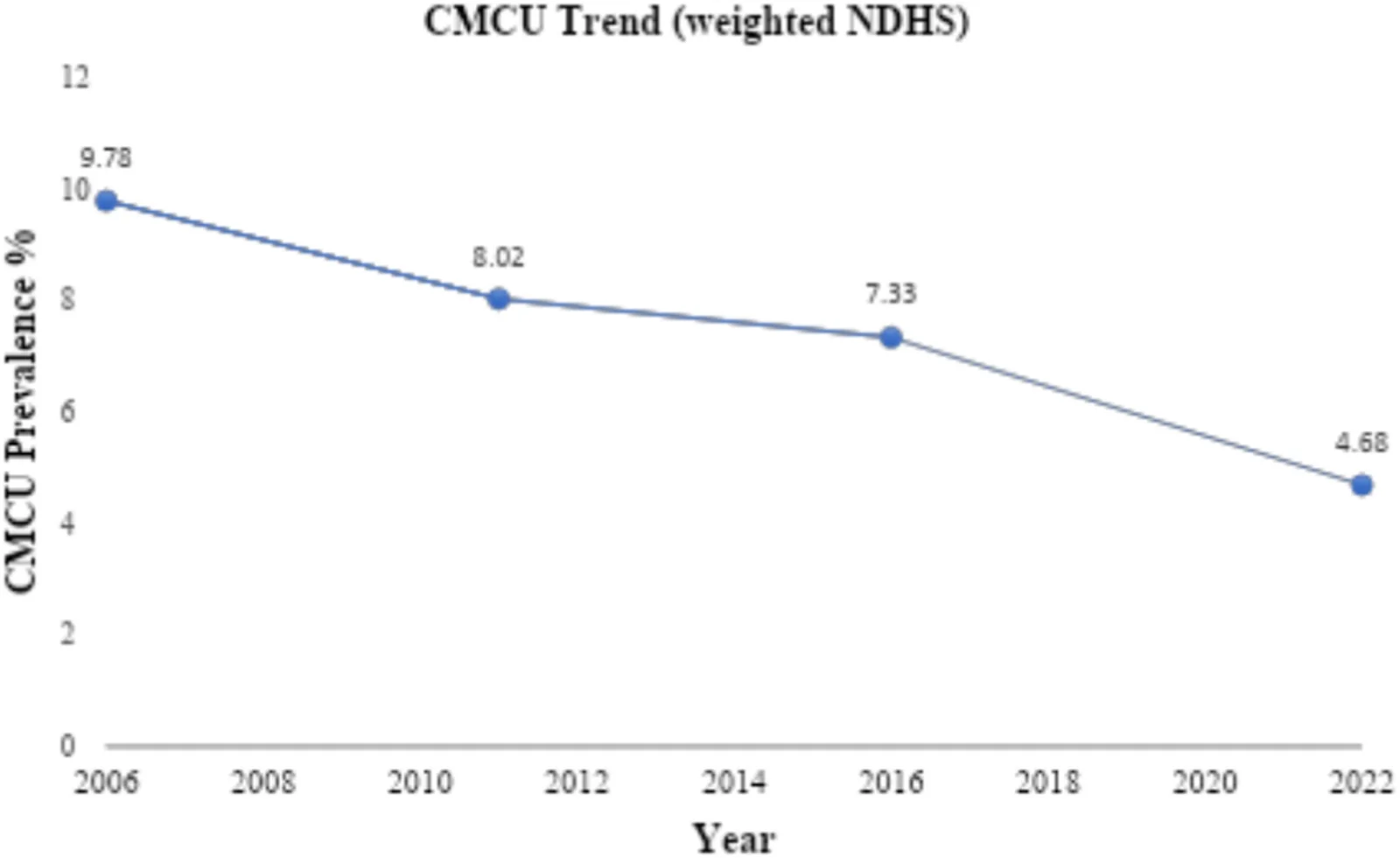
Trends in prevalence of CMCU from 2006–2022

The data from four NDHS showed the decline in the prevalence of CMCU from 9.78% in 2006 to 4.68% in 2022, reflecting a 52.1% decrease in the course of 16 years (Fig. 2).

The pooled sociodemographic characteristics of 12,513 mother–child pairs from four cycles of NDHS (2006, 2011, 2016, and 2022) are summarized in Table 2. The child’s sex was almost equally distributed (51.4% male and 48.6% female), as well as the age of children were proximate across five categories. The mother’s age majorly lies at 25–34 years (47.7%), with no formal education (44.7%), secondary education and above (32.8%). Almost all mothers were married (99.0%) and mainly employed (59.6%). Most of settlement (69.7%) was rural, the households were majorly male-headed (74.0%), > 5 family members (53.8%), and poor in wealth index (45.3%). On the other hand, most births occurred at home (60.9%), normal vaginal deliveries (93.3%), unimproved toilet (53.9%), and source of drinking water without a pipe (60.7%). Despite all these adverse scenarios, the majority of mothers (84.2%) availed antenatal care at least once. Most births were singleton (99.0%) and order of birth is 1–3 (78.9%).

**Table 2:** Sociodemographic characteristics of mothers with children in Nepal NDHS 2006–2022

Characteristics	2006–2022 *N* (%)
Sex of child	
Female	6,066 (48.61)
Male	6,447 (51.39)
Current age of child (months)	
0–11	2,354 (18.91)
12–23	2,505 (19.93)
24–35	2,608 (20.95)
36–47	2,543 (20.27)
48–59	2,503 (19.95)
Mother’s age (years)	
15–24	5,188 (41.28)
25–34	5,941 (47.67)
35–49	1,384 (11.05)
Maternal education level	
No education	5,594 (44.68)
Primary education	2,766 (22.52)
Secondary and above	4,153 (32.80)
Maternal work status	
Employed	8,014 (59.61)
Unemployed	4,499 (40.39)
Marital status	
Married	12,394 (99.04)
Unmarried	119 (0.96)
Place of residence	
Rural	8,228 (69.73)
Urban	4,285 (30.27)
Number of household members	
5 or fewer	5,796 (46.20)
More than 5	6,717 (53.80)
Number of children under 5	
2 or more	5,434 (42.51)
Less than 2	7,079 (57.49)
Sex of household head	
Female	3,338 (26.04)
Male	9,175 (73.96)
Wealth index	
Middle	2,380 (21.39)
Poorer	2,634 (21.27)
Poorest	3,686 (24.07)
Richer	2,168 (18.76)
Richest	1,645 (14.51)
Place of delivery	
Health facility	4,467 (39.07)
Home	7,003 (60.93)
Mode of delivery	
Cesarean section	656 (6.67)
Normal delivery	10,814 (93.33)
Type of toilet facility	
Improved	5,878 (46.10)
Unimproved	6,635 (53.90)
Source of drinking water	
Non-piped	7,088 (60.67)
Piped	5,425 (39.33)
Number of antenatal visits	
No	1,496 (15.82)
Yes	7,609 (84.18)
Birth order	
1–3	9,794 (78.87)
> 3	2,719 (21.13)
Birth type	
Multiple birth	127 (0.96)
Single birth	12,386 (99.04)

Table 3. The comparison statistics of the models of multilevel logistic regression of coexisting maternal and child undernutrition. Model I (empty model); Model II (model with individual-level variables); Model III (model with community-level variables); Model IV (full model). ICC = intra-class correlation; PCV = proportional change in variance; MOR = median odds ratio; AIC = Akaike Criteria; BIC = Bayesian Information Criteria. The final model, Model IV (full model), showed the best relative fit (lowest AIC = 3285.29; highest log-likelihood = −1600.65) against all the candidate models. With individual and community-level covariates in the model, the Clustering and Linear Regression of communities was 80.2% (PCV = 0.802). A PCV of 0.802 in Model IV explains 80% of the community level variation has been explained after adding both the individual and community factors. Even so a low ICC = 0.029 in Model IV shows that only 2.9% of the remaining, unexplained variances in Child and Maternal Co-undernutrition (CMCU) is caused by unmeasured community clustering factors as compared to ICC = 0.134 in model I which shows that 13.4% of the variance is explained by unobserved community level factors. This means Model IV shows that 97.1% variance is explained by individual and household level factors.

**Table 3:** Measures of variation and model comparison for CMCU in Nepal across four models

	ICC	PCV	MOR	AIC	BIC	LogLik
Model I	0.134152	0	1.975904	6648.367	6663.217	−3322.18
Model II	0.053244	0.637023	1.507273	3290.161	3491.862	−1615.08
Model III	0.097461	0.303039	1.765703	6558.509	6662.457	−3265.25
Model IV	0.029761	0.802024	1.35394	3285.293	3567.675	−1600.65

The presence of multicollinearity among the independent variables in Model IV was evaluated with Generalized Variance Inflation Factor (GVIF). For categorical independent variables with more than one Degree of freedom (Df), the adjusted $\:\left(GVI{F}^{1/\left(2\times\:Df\right)}\right)$ was interpreted. The adjusted GVIF values of the independent variables ranged from 1.01 to 1.43 and were lower than the common threshold value of 5. No strong collinearity was observed in the independent variables. Therefore, all the variables in Model IV were included for the multilevel analysis (Table 4).

**Table 4:** Multicollinearity testing using VIF

CATEGORY	GVIF	Df	GVIF^(1/(2*Df))
Age of Respondent	1.934369	2	1.179328651
Education	2.078905	3	1.129724205
Wealth Status	2.884301	4	1.141576638
Child Age in Months	1.385853	4	1.041632821
Child Sex	1.018857	1	1.009384659
Antenatal Visits	1.224238	1	1.106452782
Media Exposure	1.147837	1	1.071371673
Cigarette Smoking	1.179643	1	1.086113608
Type of Toilet Facility	1.553658	1	1.246458275
Contraceptive Use	1.25593	1	1.120682786
Births In Last 5 Years	1.181088	1	1.086778656
Birth Order Number	1.803985	1	1.343125183
Birth Interval	1.159451	1	1.076777857
Type of Birth	1.063076	1	1.031055728
Residence	1.258483	1	1.121821279
Province	1.886519	6	1.054318359
Family Size	1.316356	1	1.147325466
Source of Drinking Water	1.349382	1	1.161629123
Electricity	1.221486	1	1.105208419
Employment Status of Respondent	1.24477	1	1.115692641
Pregnancy Status	1.181309	1	1.086880364
Age Gap	1.011005	1	1.005487619
Education Gap	1.339632	1	1.157424856
Community Distance from Health Facility	1.143057	1	1.069138388
Community Illiteracy Level	1.69227	1	1.300872909
Community Poverty Level	2.050567	1	1.431979979

Table 5 shows the results of the multivariable logistic regression of the individual, household and community-level factors associated with the study outcome as adjusted odds ratios (AOR) with 95% confidence intervals (CIs). The results are derived from four model specifications.

**Table 5:** Multilevel logistic regression analysis result of coexisting maternal and child undernutrition in Nepal 2006–2022

Characteristics	Model I	Model II	Model III	Model IV
Age of Respondent				
15–24		1		
25–34		1.00 (0.83–1.21)		
35–49		1.17 (0.87–1.57)		
Education				
No Education		1		1
Primary		0.77 (0.63–0.94)*		0.73 (0.60–0.90)**
Secondary		0.43 (0.34–0.56)***		0.42 (0.32–0.54)***
Higher		0.22 (0.12–0.42)***		0.22 (0.12–0.42)***
Wealth Status				
Poorest		1		
Poorer		0.99 (0.81–1.20)		
Middle Income		0.97 (0.78–1.21)		
Richer		0.87 (0.67–1.12)		
Richest		1.04 (0.73–1.47)		
Child Age in Months				
< 12		1		1
12–23		1.83 (1.41–2.36)***		1.86 (1.44–2.40)***
24–35		2.02 (1.56–2.62)***		2.13 (1.67–2.73)***
36–47		1.78 (1.34–2.36)***		1.96 (1.52–2.52)***
48–56		1.65 (1.23–2.21)***		1.85 (1.43–2.38)***
Child Sex				
Male		1		
Female		1.03 (0.90–1.19)		
Antenatal Visits				
No ANC		1		
Had ANC		1.03 (0.81–1.31)		
Media Exposure				
No		1		1
Yes		0.74 (0.61–0.89)**		0.76 (0.64–0.92)**
Cigarette Smoking				
No		1		
Yes		1.14 (0.92–1.42)		
Type of Toilet Facility				
Unimproved		1		1
Improved		0.72 (0.60–0.86)***		0.72 (0.60–0.86)***
Contraceptive Use				
Do Not Use		1		1
Use		0.70 (0.60–0.83)***		0.71 (0.61–0.83)***
Births In Last 5 Years				
< 3 Births		1		1
> 3 Births		0.77 (0.57–1.05)		0.82 (0.61–1.10)
Birth Order Number				1
< 3 Birth Order Number		1		
> 3 Birth Order Number		1.07 (0.87–1.31)		
Birth Interval				
< 36 Months		1		1
> 36 Months		0.88 (0.74–1.05)		0.90 (0.76–1.06)
Type of Birth				
Single		1		1
Multiple		1.91 (1.09–3.36)*		2.05 (1.17–3.58)*
Age Gap				
< 5 years		1		1
> 5 years		0.31 (0.07–1.40)		0.31 (0.07–1.39)
Education Gap				
Same/Less		1		1
Mother More		1.28 (0.99–1.66)		1.27 (0.99–1.64)
Family Size				
> 5 members		1		
< 5 Members		1.02 (0.88–1.19)		
Employment Status of Respondent				
Not Employed		1		
Employed		1.09 (0.92–1.29)		
Pregnancy Status				
Not Pregnant		1		
Pregnant		1.01 (0.78–1.30)		
Residence				
Urban			1	1
Rural			1.30 (1.08–1.57)**	1.11 (0.92–1.34)
Province				
Bagmati			1	1
Gandaki			0.88 (0.61–1.28)	0.98 (0.67–1.42)
Karnali			0.82 (0.60–1.12)	0.86 (0.64–1.17)
Koshi			0.90 (0.68–1.18)	0.90 (0.68–1.19)
Lumbini			0.81 (0.61–1.09)	0.81 (0.60–1.08)
Madhesh			0.87 (0.65–1.17)	0.78 (0.59–1.04)
Sudurpashchim			0.52 (0.38–0.70)***	0.53 (0.39–0.71)***
Source of Drinking Water				
Non-piped			1	
Piped			0.94 (0.79–1.11)	
Electricity				
Available			1	1
Not Available			1.23 (1.05–1.43)**	1.11 (0.94–1.30)
Community Distance from Health Facility				
No Big Problem			1	1
Big Problem			1.40 (1.21–1.63)***	1.28 (1.10–1.49)**
Community Illiteracy Level				
Low			1	1
High			1.70 (1.41–2.05)***	1.05 (0.86–1.29)
Community Poverty Level				
Low			1	
High Poverty			1.12 (0.93–1.35)	

*Significance levels: *p* < 0.05; **p < 0.01 (highly significant); *** *p* < 0.001 (very highly significant)

Model I consisted of individual and household-level demographic, socio-economic and reproductive characteristics. Model II assessed household infrastructure and contextual factors (i.e. type of residence, province, main source of drinking water, presence of electricity in the household). Model III consisted of distance to health facilities, community illiteracy and community poverty, which are only community-level factors. Model IV is the final optimized model obtained through backwards method by which we only keep significant factors at 0.2 significance level and it includes all factors that were presented in Model I, II and III together.

Model IV, which is the final model based on study utilization at 0.2 cut-off value, has the statistically significant protective effect of primary education (AOR = 0.73, 95% CI: 0.60–0.90), secondary education (AOR = 0.42, 95% CI: 0.32–0.54), and higher educational attainment (AOR = 0.22, 95% CI: 0.12–0.42). The age of the child remained significantly associated with the outcome variable and the odds ratio ranged from 1.86 to 2.13 for different age groups. Media exposure (AOR = 0.76, 95% CI: 0.64–0.92), access to improved toilet facility (AOR = 0.72, 95% CI: 0.60–0.86), and contraceptive use (AOR = 0.71, 95% CI: 0.61–0.83) remained statistically significant as protective factors. Multiple birth was found to have statistically significant higher odds (AOR = 2.05, 95% CI: 1.17–3.58) for the outcome variable. The association of living in rural areas were found to be non-significant after controlling for individual and community factors, however, the protective effect of residing in Sudurpashchim province remained statistically significant (AOR = 0.53, 95% CI: 0.39–0.71). Community distance to health facility remained as statistically significant risk factor (AOR = 1.28, 95% CI: 1.10–1.49) along with community illiteracy. However, the effect of community illiteracy was significantly reduced and found to be non-significant (AOR = 1.05, 95% CI: 0.86–1.29). The association of electricity access also remained non-significant in the final model (AOR = 1.11, 95% CI: 0.94–1.30).

The spatial clustering across survey years was assessed by using K-nearest neighbors spatial weights matrix (k = 8) based Moran’s I spatial autocorrelation statistic to determine CMCU clustering pattern in space over time. The results are presented in the Fig. 2. In the survey years 2006, 2011, 2016, and 2022 findings show a gradual shift from weak-no statistically significant spatial autocorrelation from 2006 to 2016, to positive statistically significant spatial autocorrelation in year 2022. The Moran’s I result for 2006 stand at 0.034 (Z = 1.322), indicating weak positive autocorrelation, however this was not statistically significant. In the following survey year 2011, the value decreased to 0.022 (Z = 0.874) and in 2016 0.018 (Z = 0.847), indicating statistically random pattern of CMCU across Nepal. The Moran’s I statistic value almost tripled to 0.058(Z = 2.548) in the year 2022 and this was found to be significant at 5% probability level. The results overall imply weak-no statistically significant spatial clustering of CMCU in 2006, a statistically random pattern in 2011 and 2016, and positive statistically significant spatial clustering of CMCU in 2022.

This shows the weakening of the effect of distance over time, as the burden of coexisting undernutrition is found concentrated along certain geographical locations in the later years. The result also reveals the growing effects of location-based effect and allocation of subnational focus in explaining residual burden of CMCU, evident in the later years of Nepal’s nutrition transition.

**Fig. 3:**
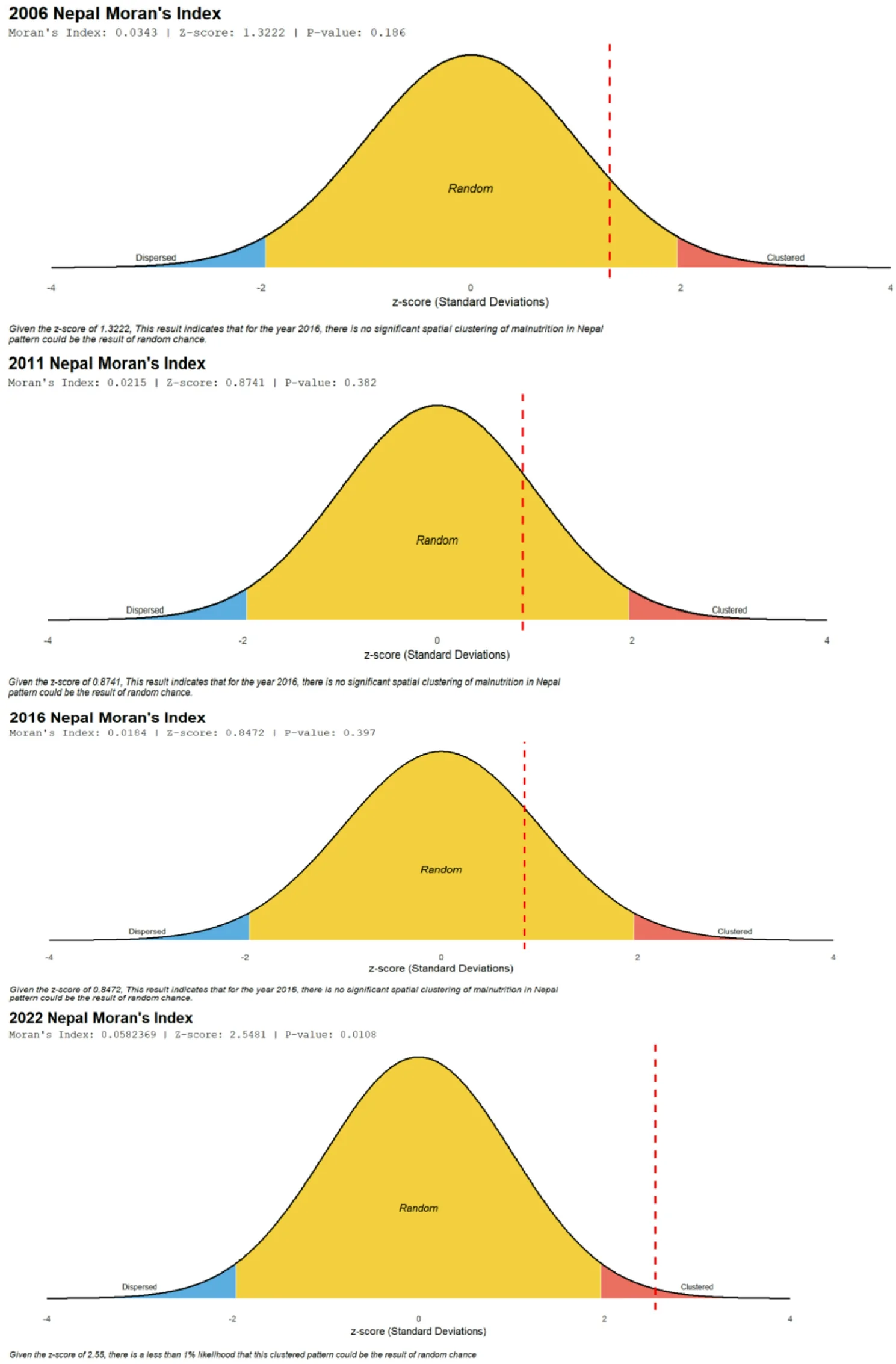
Spatial Clustering of coexisting maternal and child undernutrition in Nepal from 2006–2022

The spatial distribution of coexisting maternal and child undernutrition (CMCU) in Nepal, by province, according to NDHS (2006, 2011, 2016, and 2022) is showed in the Fig. 3. The CMCU displayed significant spatial diversity among provinces and a decrease in its burden over time. In the year 2006, Bagmati Province registered the most elevated CMCU prevalence at 12.7%, followed by Karnali Province (11.2%), Gandaki Province (10.0%), and Madhesh Province (9.8%). These figures indicate a burden higher than its average for most central and western provinces. The CMCU was also most prevalent in 2011, and despite its decline by the general average of the survey year, the highest prevalence was registered in Karnali Province (11.6%). There was, however, a significant drop in CMCU prevalence in 2016 that continued across most provinces, with Madhesh Province and Karnali Province recording the highest figures, at 8.9% each, followed by Koshi Province (7.4%). Then, in 2022, the CMCU prevalence had dropped in all provinces, with Madhesh Province, at 7.7%, recording the highest. Meanwhile, although Sudurpashchim Province exhibited the second-lowest prevalence, it also displayed a significant drop at 2.4%.

**Fig. 4:**
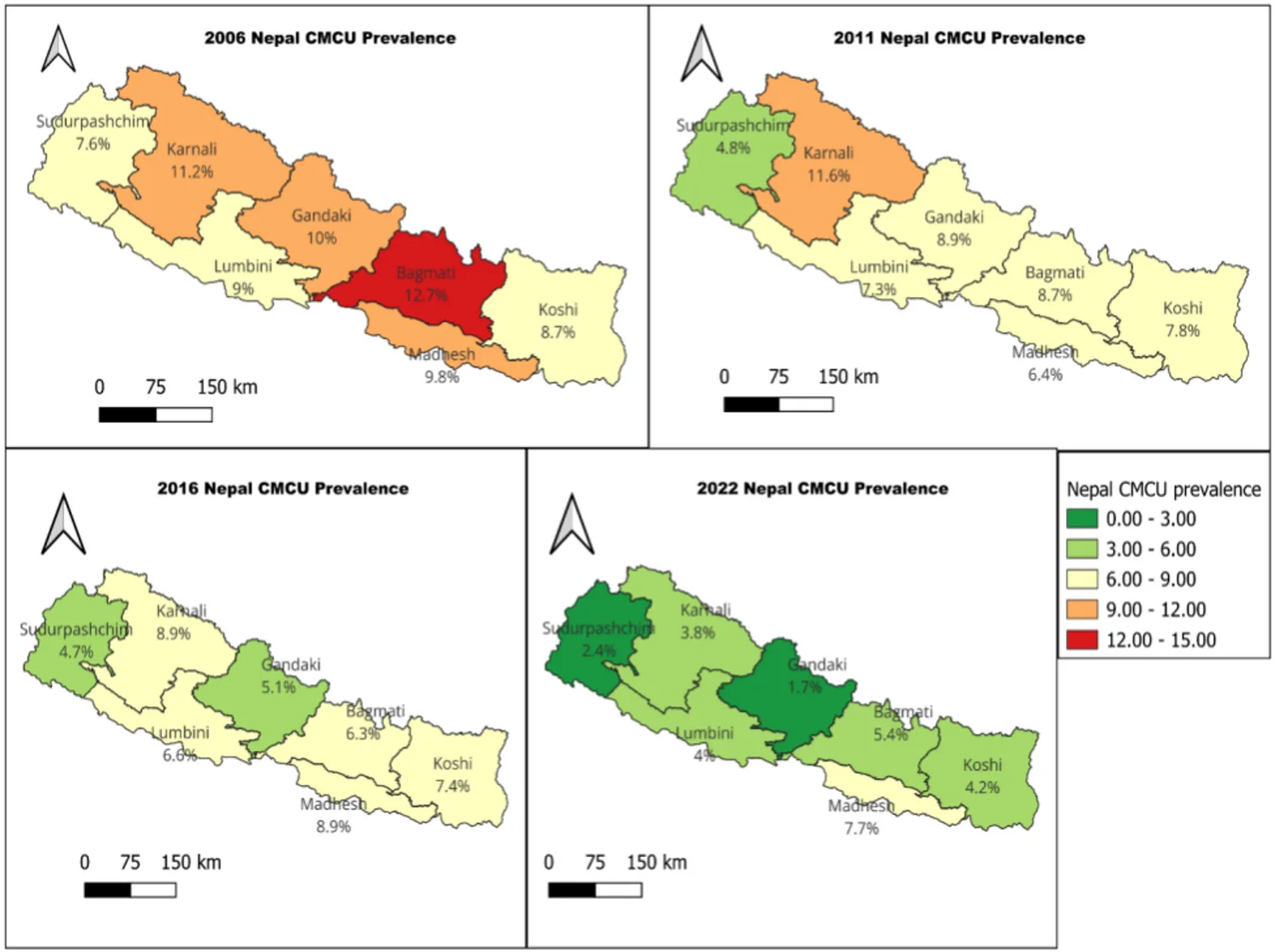
Nepal CMCU prevalence from 2006–2022

Overall, the map’s spatial distributions showed a declining trend in the CMCU burden across the surveyed years. However, a significant spatial disparity remained evident in the surveyed CADs, with Madhesh Province recording the highest prevalence for most years and Karnali Province also revealing relatively high figures compared to major provinces (Fig. 4).

Getis-Ord Gi* hotspot analysis was applied to determine significant CMCU high (hotspots shown in red) and low (cold spots shown in blue) prevalence clusters at the cluster level for all four survey rounds. Non-significant clusters are represented in white in the map. The hotspot analysis demonstrates the significant change in CMCU geographical diffusion throughout the study period (16 years).

In 2006, hotspots were majorly concentrated in the Terai belt of southern lowlands, especially in the eastern and central regions that share borders with India. The cold spots were present in the mid-hill as well as mountainous areas of the Gandaki and western part of Nepal. This trend continued in 2011 as hotspots were still clustered in the eastern and central Terai while cold spots thrived in the far-western and central hill areas. By 2016, hotspots turned increasingly fragmented and dispersed as further high-burden areas started to emerge in the isolated areas of the mid-western and far-western regions. Cold spot areas remained confined to the northern-central hill regions and uppermost mountain regions.

In 2022, the hotspots became denser and clustered into a specific region, primarily located in the southern plains of Madhesh Province and neighboring districts of Lumbini and Koshi provinces, making it a proximate belt of significant coexistence of undernutrition. The coldspots became denser and clustered into a specific region, primarily the northern hill and mountain districts of the Sudurpashchim, Karnali, and Gandaki provinces. The differences between hotspot and cold spot regions showed greater statistical significance in the year 2022 than any previous survey round, which also supports the significant value of Global Moran’s I (0.058; Z = 2.548) result, suggesting that CMCU is now geographically clustered as compared to the previous survey years which showed clusters as geographically dispersed.

**Fig. 5:**
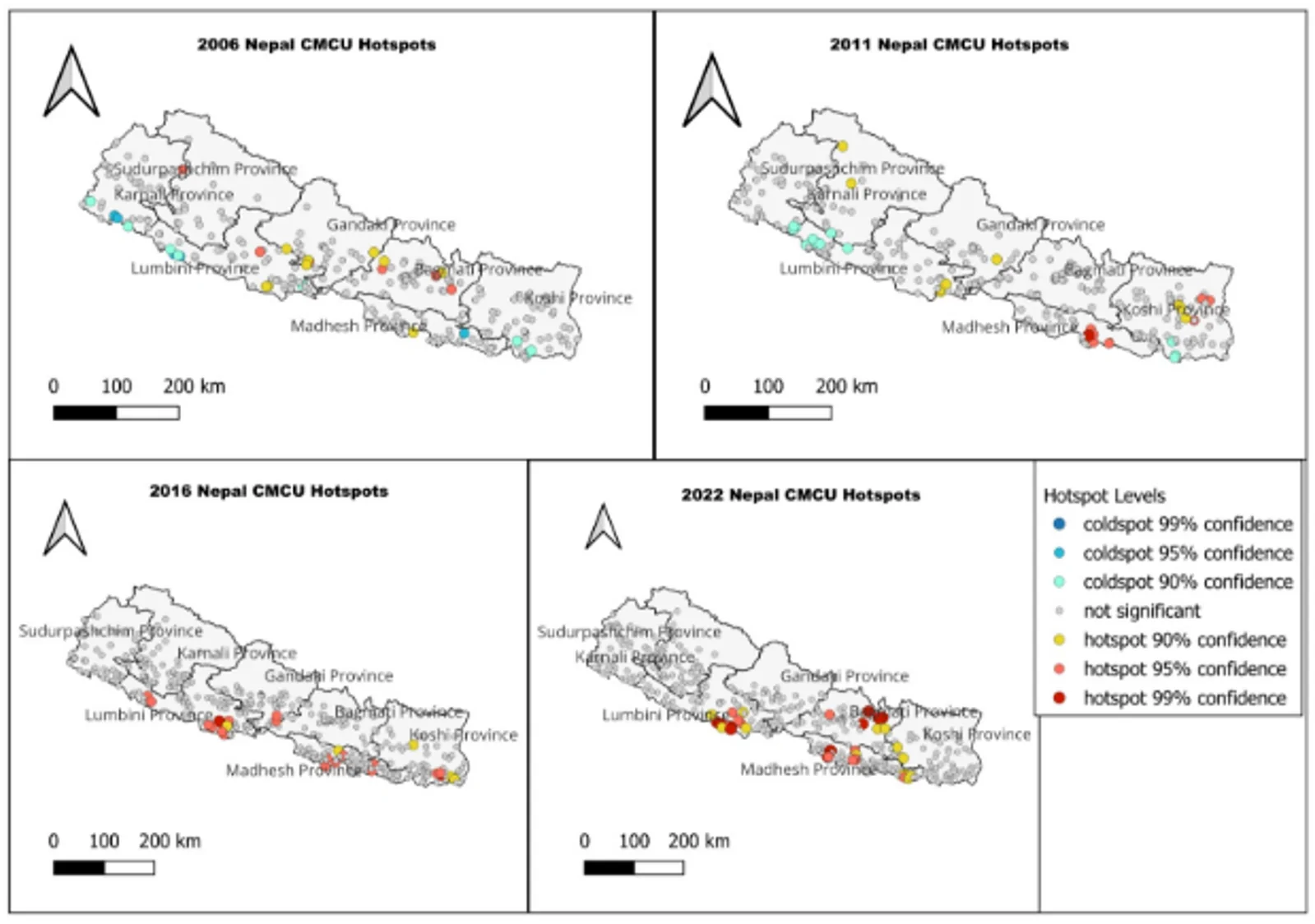
Hotspot analysis result of CMCU across regions in Nepal based on NDHS 2006–2022 data

The increasing significance of location-specific factors and tailored interventions at the subnational level for addressing the associated condition of maternal and child undernutrition in Nepal could be evidenced by the rising geographical clustering of CMCU, from Terai and adjacent lowland regions, as well as the emerging cold spots in the northern hill and mountain regions (Fig. 5).

## Discussion

This paper reviews the progress in CMCU in Nepal from 9.78% in 2006 to 4.68% in 2022 or about 52.1% reduction in 16 years. The finding is relevant particularly for South Asia which topped the stunting (33.2%) and wasting (14.8%) rates internationally with no country on track to achieve Sustainable Development Goals 2 (Nutrition Targets) [26]. Most countries of South and Southeast Asia have recorded lower CMCU, but Nepal follows the stories of success alongside Bangladesh and Cambodia, where national nutrition programs resulted in decreased child undernutrition [27]. In addition, CMCU in Nepal increases credentials of the country which holds the record of the fastest child stunting prevalence decline globally from 2001 to 2011 at 56.6% to 40.0% [28].

This progress in prevalence is coincided with the scale-up of community-based nutrition programs, strengthened maternal and child health services, and the Multi-Sectoral Nutrition Plan (MSNP) implementation [26]. MSNP is among the first multi-sectoral governments’ effort to tackle undernutrition and malnutrition problems through sectors collaboration which includes health, agriculture, education, WASH, and social welfare [29]. The MSNP’s first lapse was in 2013, followed by MSNP-III proposed for 2023 to 2030. The decline in CMCU coincided with implementation of these programs, which makes it plausible that these factors contributed, but it cannot be concluded causally in a cross-sectional sample. Suaahara’s project was implemented from 2011 to 2023, covering 42 out of 77 districts in Nepal. The integrated nutrition, health, family planning, WASH, and agriculture project was implemented through social and behavior change communication (SBCC) approach, using interpersonal communication, community mobilization, mass media programs (Bhanchhin Aama radio program), and SMS [30, 31]. Process evaluations of Suaahara showed that there was improvement of maternal and child nutrition knowledge and practices. In comparison, program districts and neighborhood households’ less-favored status and non-neighbour households’ gap was smaller than control districts [32]. Another program which contributed to Nepal community-based nutrition program is the Female Community Health Volunteers (FCHVs) program, approximately 50,000 are stationed across the country. It is the forefront for community-based nutrition interventions, such as vitamin A supplementation, iron-foliate supplementation distribution, nutrition counselling, in the remote and geographically isolated areas [26, 33].

Maternal education was the strongest protective factor against CMCU in our study. In the fully adjusted model, the odds were 80% lower for mothers with high education compared to never attended (AOR = 0.20, 95% CI: 0.07–0.58). The odds were 68% lower with primary education and 39% lower with secondary education. Many studies have shown statistical significance for various forms of malnutrition, including CMCU. Hong et al. (2025) in a pooled analysis of seven South and Southeast Asian countries highlighted the importance of maternal education showing statistically significant results at -log odds ratios across six countries with high maternal education as a protective factor against coexisting forms of malnutrition among children. In a decomposition analysis of inequalities in childhood malnutrition in 27 countries, maternal education was highlighted as an explanatory variable significantly influencing the gap between educated and uneducated mothers. In the case of Nepal, the malnutrition gap was smaller for children born from educated mothers attributed to compositional effects as maternal education [34]. The mechanisms through which maternal education influences child nutrition have been well-discussed. Mothers with a higher education level reportedly seek antenatal and postnatal care services, practice health recommended behaviors including exclusive breastfeeding, appropriate complementary feeding and utilization of skilled birth attendance [35]. Education-buffered health literacy, allows mothers to better process nutrition messages relayed through Suaahara interventions channels including Bhanchhin Aama program, radio program and community mobilization activities [30].

In addition, education positively influences women’s marriage and childbearing age, women’s empowerment in family decision-making and economic opportunities, contributing to improved nutrition of the children in the family [36].The inter-generational effect of maternal education is more pronounced than other impact pathways.

Maternal short stature is the definite correlate of undernutrition in children in South Asia, which is used as an indicator for early life undernutrition. In Bangladesh, children of short-statured mothers (under 145 cm) had nearly double risk of stunting than, and more than triple risk of severe stunting than those of tall mothers [19]. In a pooled study from 54 low-middle-income countries (LMIC), the relative risk (RR) of stunting was found to be greater than two times (RR = 2.132, 95% CI: 2.103, 2.161) for children of short mothers compared to tall mothers [37].

Children aged 12–59 months had higher CMCU odds compared with children aged less than 12 months, and was highest among children aged 24–47 months (AOR = 2.14, 95% CI: 1.55–2.96) reflecting the fact that the complementary feeding period (6–23 months) is a nutritionally vulnerable time period and the time when much of the stunting occurs in South Asia. Aguayo et al., reported that in South Asia, an estimated 37% (~ 64 million) of children under five years old are stunted and much of the stunting occurs during the complementary feeding period [26].Minimum meal frequency and minimum diet diversity were both positively associated with height-for-age in 6–23 month aged children in Nepal, and the combination of these indicators had the greatest influence on child growth outcomes [26]. The higher odds in older children reported in this study may also relate to the shift from exclusive breastfeeding to family foods, which in Nepal are often primarily cereals with limited micronutrient density and high inaccessibility to animal-source foods. The Suaahara program specifically addressed poor complementary feeding practices through the Social Behavior Change Communication (SBCC) platforms used by the program, promoting eggs and animal-source foods, and enhancing the use of orange-fleshed vegetables in the diet of children [38].The fact that CMCU odds remained significantly greater among children 12–59 months suggests that further attention may be warranted on improving complementary feeding practices, especially among poorer households. More targeted age-specific nutrition may warrant attention in future interventions efforts during the 1000-day window, and especially during the 6–24 months transition period when children are very vulnerable to stunting.

The use of contraceptive was linked with 25% lesser odds for CMCU in both the individual level model (Model II) and the fully adjusted model (Model IV). This is consistent with the existing evidence about the association of family planning on child nutrition. Planned pregnancies with suitable timings allow mothers to recover physically and nutritionally between pregnancies. This improves the maternal health and nutritional status of average for next child [36]. As reported in a study based on NDHS 2016, the highest percentage of stunting (52.7%) and underweight (41.1%) was recorded at children with birth order > 3 and birth interval < 2 years in Nepal. 12–24 months after marriage was optimal timing to delay child-bearing to avoid stunting (OR 0.6, 95% CI 0.4–0.9) [2]. The favorable influence of contraceptive use on CMCU is thought to act via diverse pathways. Firstly, the time and energy devoted to each child is greater due to longer birth intervals. This translates to longer exclusive breastfeeding duration and better complementary feeding. Secondly, family planning averts maternal depletion syndrome caused by close-timed pregnancies that use up maternal nutrient reserve, leading to low birth-weight and subsequent undernutrition. Lastly, contraceptive use is associated with maternal empowerment and healthcare seeking tendencies such as antenatal care attendance and institutional delivery [36]. Family planning can potentially be a high-impact sector to synergize with nutrition in the undernutrition and reproductive health nexus as seen in Suaahara. The expected and insightful finding is the attenuation of odds ratios from Model II to Model IV. For example the adjusted odds ratio of contraceptive use goes from AOR = 0.70 in Model II to AOR = 0.71 in Model IV and the adjusted odds ratio of media exposure is AOR = 0.74 in Model II to AOR = 0.76 in Model IV. By confounding structure, when the individual-level socioeconomic factors (i.e., maternal education and wealth) are included in the model, the protective effects of proximal determinants (e.g., contraceptive use and media exposure) are reduced. The results successfully replicate confounding structure. The socioeconomic status of the subjects impact the exposure (i.e., contraceptive use and media access) and the outcome (CMCU). The reduction in effect in Model IV indicates that these proximal determinants do not function as protective determinants independently. Rather, they operate sometime through a socioeconomic pathway.

In the community level, the only contextual factor that still remained significant in the full model (Model IV) was perceived distance to the health facilities. Individuals who perceived distance to the health facilities as a “big problem” were 24% more likely to have CMCU (AOR = 1.24, 95% CI: 1.00–1.52). This result is also in line with the most recent geospatial literature on Nepal’s health system. A 2022 study utilizing the Nepal Health Facility Registry for high-resolution mapping found the geographical accessibility index of maternal health services was better in the access of population in the Terai belt, central and southern sections, as opposed to the northern mountainous region. Population-weighted average travel times for antenatal care (ANC), delivery facility care, and postnatal care (PNC) were less than 30 min for 26.74 min, 40.72 min, and 39.09 min, respectively. However, less than half of the population (48.85%) enjoyed accessibility to delivery-care facilities at travel times below recommended levels (under 30 min). Karnali Province had the lowest access (35.63% under the half-hour travel time) [39].The study quantifying the impact of geographical accessibility on Maternal Healthcare-Seeking Behavior (MHSB) revealed an inverse correlation to MHSB in Nepal. The Bayesian geostatistical models concluded a unit increase in access (travel time) negatively multiplied the odds of more than four ANC visits (odds ratio (OR) 0.76, 95% BCI 0.65–0.90), delivery at health institution (OR 0.76, 95% BCI 0.64–0.90), and post-delivery mother healthcare follow-ups (OR 0.87, 95% BCI 0.76–0.99) [39]. “Too far/no transportation” questions were also amongst the reasons cited in the 2022 Nepal Demographic Health Survey (NDHS) for not seeking maternal health services, and are more pronounced in Karnali Province [39]. Overall, it must be posited that despite the progressing expansion of healthcare facilities in Nepal, the resulting access of marginalized communities still limits MHSB, as physical access is a stumbling block for those in remote mountainous or far-off communities. It is also notable how the other community-level effects have been attenuated in Model IV. Rural residence, which had 29% increase defined odds of CMCU in Model III (adjusted odds ratio AOR = 1.29, 95% confidence interval (CI): 1.07–1.56) was not significant in the fully-adjusted model (AOR = 1.05, 95% CI: 0.81–1.35), which means lower MSHB is no more a rural phenomenon. In this same factor, rural-urban differences in CMCU are accounted for by the individual socioeconomic-level factors co-determined in the model. Other community-level factors such as illiterates and the poor, which were significant in Model III, were shown no effect in Model IV [40]. These same factors have been shown in similar multilevel analyses in other communities, where individual family-level factors are contributory to MSHB clustering more than contextual factors inherent to the community. The result is that the clustering of households with MSHB is more dependent on the geographic closeness of disadvantaged households.

For the spatial analysis, it was observed that over the years, there was a paradigm shift in the geographical patterning of CMCU. The years, 2006–2016, the spatial autocorrelation was weak or was random, while the survey of 2022 observed strongly positive spatial clustering (Moran’s I = 0.058, Z = 2.548). The hotspots for the CMCU were in the southern Terai provinces, namely, Madhesh, Lumbini and, Koshi. The noticeable change of clustering in 2022 versus dispersed in earlier years indicated that the CMCU was clustering more within geographical space, which implies the geographical inequity has escalated at this level of hierarchy despite the positive national level change. The clustering of CMCU in the Terai belt as observed is not surprising since previous spatial analyses of child undernutrition also found it in the southern provinces of Nepal. Based on the Bayesian distributional bivariate probit model, children from the Terai districts of Madhesh Province and Koshi Province were found to be more likely to be wasted and wasting was highest at 18.9% among children of the Eastern Terai as compared to all other regions [33]. The associated wasting hotspots in Eastern Terai can be associated with high infection rates and low energy intake in the context of the southern plain’s livelihood and environmental conditions [33]. The cold spots in the hill and mountain districts of northern Sudurpashchim, Karnali, and Gandaki provinces can suggest certain improvements in CMCU in these provinces, which can be a result of specific targeting of some interventions and out-migration of the healthier population. The observed provincial inequalities in this study are similar to the previously identified inequalities in health and development in Nepal. In the context of children under-five years old, Karnali Province is characterized by the highest level of stunting nationally from 2001 to 2016, being 70.5% in 2001, and 54.4% in 2016, while the highest nationally predicted reduction was found in Bagmati and Gandaki provinces [41]. In the context of under-five mortality nationally, the highest prevalence is seen in Karnali Province (54.3 per 1000 live births for males) and Sudurpashchim Province (47.5 for females), while the lowest and most improved levels are in Gandaki Province (25.4 for males, 21.2 for females) [42]. Some structural factors that are responsible for the inequities are food insecurity, poor road access, low market access, and high concentration of marginalized ethnicities and castes [33, 40]. It creates a need to focus resources geographically. The stark CMCU hotspots that emerged in 2022 highlights the inadequate effectiveness of national policies, and the need for subnational strategies to account for context [40]. The Terai context impacts geography accessibility and sociocultural aspects (including health access barriers). Given the entry of health federalism in Nepal after 2015, where health service delivery was devolved to provincial and local governments, the opportunities for targeted programming exist. However, provincial and local governments require better capacity to manage the technical demands and media of targeted nutrition programming [43].

The protective effect of media exposure in Model II (AOR = 0.74, 95% CI: 0.58–0.95) was not significant in the completely adjusted Model IV (AOR = 0.79, 95% CI: 0.62–1.01). This reflects the notion that the protective effect occurs via the socioeconomic characteristics that influence both media exposure and nutrition. This corresponds to the evaluation of SBCC platforms in Suaahara where mass media exposure (especially Bhanchhin Aama radio program) was positively associated with improved dietary diversity of maternal and child diet, though the effect was influenced by household wealth and maternal education [30]. This suggests that media exposure alone was not enough to overcome the covariates that affect both media effect and nutrition, thus, antenatal interventions also need to consider the structural determinants of nutrition such as poverty and education. While the Suaahara experience showed the potential of SBCC in Nepal, it also documented the limitations. A dose-response analysis showed that exposure to multiple SBCC platforms such as interpersonal communication, community mobilization, and mass media had a positive association on dietary diversity. Exposure to all the three platforms had 2.33 times the odds of attaining minimum dietary diversity compared with no exposure [31]. It also documented that FCHV workload was a barrier for effective coverage in remote areas, thus the peer facilitator model was needed to be piloted [33]. The peer facilitator model of training done in disadvantaged villages of Bajhang, Bhojpur, and Rupandehi districts showed modest but significant improvement in knowledge and practices around maternal and child nutrition, including dietary diversity [33].Based on this evidence, the future CMCU interventions could implement communication strategies that are layered with mass media and community-based interpersonal communication formats and ensure equal standards of reach across socioeconomic groups.

The outcomes of the study provides the evidence for action for the implementation of MSNP-III (2023–2030) that intends the model of scaling up from the national to 753 local government units with the end goal of eliminating malnutrition and its economic consequences to human capital development [44].Based on the results, four areas are identified as potential priorities in which first maternal education should continue to be at the forefront of nutrition policy. Investment in education especially higher education on CMCU is so large that making investment in girl’s education may contribute for improving CMCU because it is also the major proven nutrition intervention. The UNICEF conceptual framework on maternal and child undernutrition identifies women’s education and status as underlying determinants of nutritional outcome [1].

Second, family planning should be integrated with nutrition services. Based on this study, contraceptive use also protects CMCU, and the effects of birth intervals on child growth are documented [5], hence, enhancing family planning services within nutrition services is justified. Suaahara’s provision of family planning services within its multisectoral package is an example of this [32].Third, geographic targeting should be prioritized. The 2022 hotspots of CMCU that occurred in the southern Terai provinces of Nepal, addressing malnutrition in these areas requires tailored interventions that reflect the geographic, economic, and health system differences of the plains from the hills and mountains. The MSNP-III approach of focused funding for equity in resource distribution should be put to action, i.e. ensuring that hotspot districts receive additional funds, funding for road infrastructure and transport services to reach health facilities, and more community health workers in less served wards and households [33, 45]. Fourth, the community health worker system requires innovations and investments. The FCHV program has been a leading force in the success of nutrition in Nepal but the burdening of community volunteer limits reach to remote communities [33]. The peer facilitators piloted by Suaahara could greatly increase reach [33].

## Limitations

This study has a number of strengths. The utilization of four rounds of nationally representative NDHS data (2006, 2011, 2016, 2022) has provided insights into the temporal trends and spatiotemporal analysis was possible over a period of 16 years. The multilevel logistic regression model with four-levels, provided the opportunity to examine the individual and community-level determinants concomitantly while adjusting for the clustered design of the survey. The spatial analysis using Global Moran’s I and Getis–Ord Gi* hotspot analysis provided novel evidence about the geographic clustering of the CMCU in Nepal, which had never been studied before. However, the study also has limitations. The cross-sectional nature does not allow establishing a cause-effect relationship. The associations between various determinants and CMCU could be affected by reverse causation or unobserved confounders. We opted to use maternal short stature (< 145 cm) as a proxy for maternal undernutrition rather than BMI < 18.5 kg/m2 as maternal short stature reflects the cumulative effect of maternal undernutrition throughout the life-course. While the association of undernutrition with height captures intergenerational maternal-child growth effects, it does not and cannot reflect acute maternal nutritional status. Therefore, our CMCU measure misses possible occurrences of concurrent maternal wasting or maternal acute energy deficiency. The use of listwise-deletion for missing values could have caused selection bias, if at all the missingness were associated with socioeconomic or geographic-remote status of the respondents. To create harmonized boundaries for the newly divided provinces in the subsequent rounds of surveys, we made some assumptions while mapping the districts to respective provinces which could have resulted in measurement bias in CMCU, especially among the two districts (Nawalparasi and Rukum) which were divided into two after the federal structuring in 2015.

## Conclusion

This research provides national representative evidence to the spatiotemporal trends and multilevel correlates of coexisting maternal and child undernutrition (CMCU) in Nepal covering a period of 16 years. The prevalence CMCU significantly reduced during the time period of 2006 to 2022, however, it is still a substantial public health problem in the country with significant socioeconomic and regional disparities. Among all conceivable predictors, maternal education is identified as maximum protective correlates while child’s older age, limited exposure to media, no contraceptive services, lack of availability to health facilities are associated with higher odds of CMCU. The spatial evolution of CMCU in 2022 shows significant clustering in Terai provinces illustrating the indication of spatial polarization associated with CMCU in the country.

## Supplementary Information

Supplementary Material 1.

## Data Availability

This research analyzed secondary data from nepal DHS, available at https://www.dhsprogram.com.

## Declarations

The authors declare that this research has not been published in any other journal, therefore it is declared to comply with BMC publications policy. There are no images used, or other materials which require anonymity, the questionnaire followed informed consent and anonymity.
