# Supplementary material for: Spatiotemporal trends and multilevel determinants of coexisting maternal and child undernutrition in Nepal: evidence from Nepal Demographic and Health Survey 2006–2022
Source: BMC Nutr. 2026 Aug 7;12:232. doi: 10.1186/s40795-026-01452-8 (PMC13732924; doi:10.1186/s40795-026-01452-8)

SUPPLEMENTARY MATERIALS

Supplementary Figure S1: Hierarchical DAG Mapping of Operationalized Model Predictors

All Section 5 Engineered Covariates Structured Across the Tiers of the UNICEF Conceptual Framework of Malnutrition

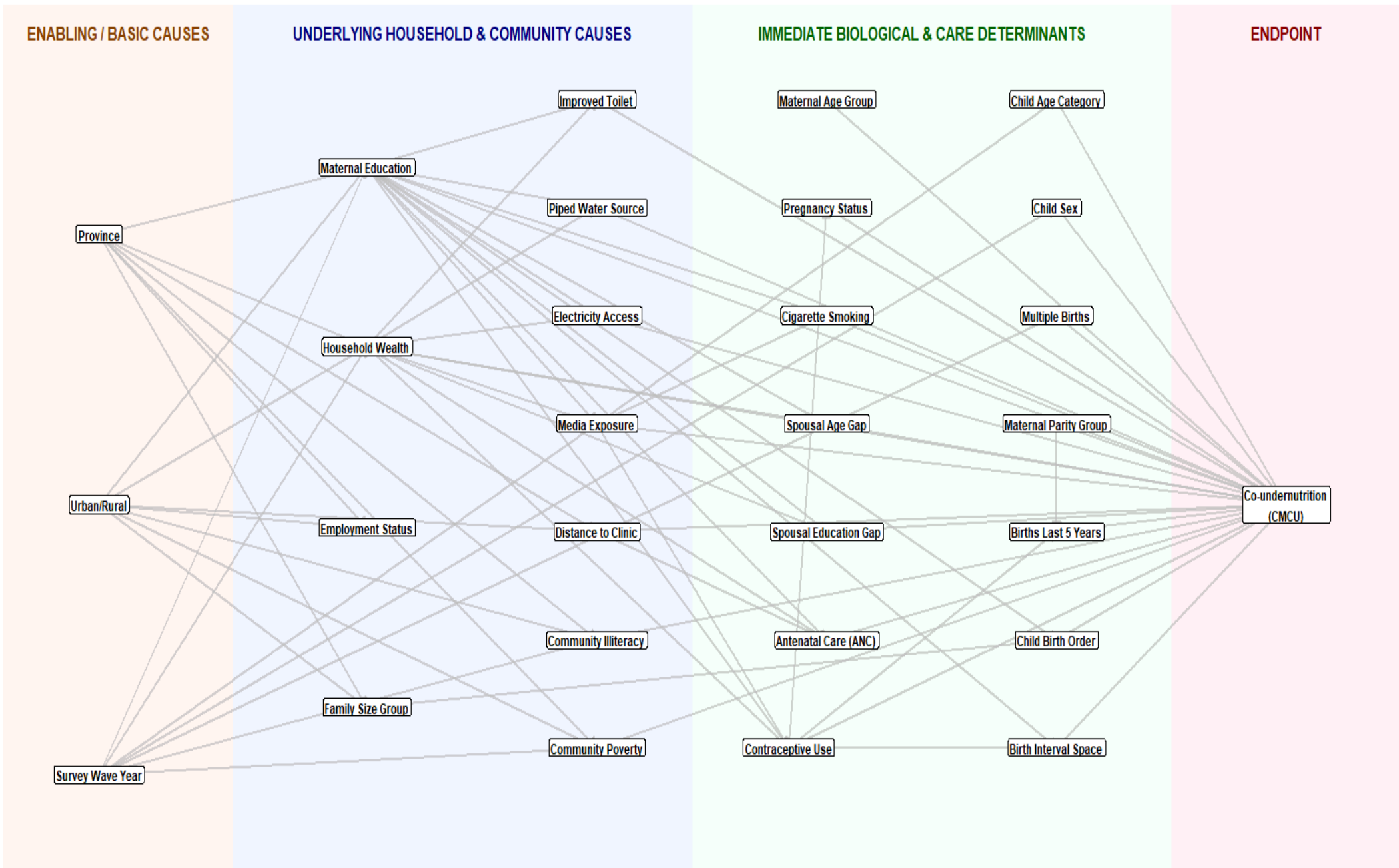

Supplement: Supplementary file 1 [file 40795_2026_1452_MOESM1_ESM.pdf]
